# Proinflammatory cytokine-induced alpha cell impairment in human islet microtissues is partially restored by dual incretin receptor agonism

**DOI:** 10.1007/s00125-025-06425-3

**Published:** 2025-05-15

**Authors:** Kristine Henriksen, Chantal Rufer, Alexandra C. Title, Sayro Jawurek, Bolette Hartmann, Jens J. Holst, Filip K. Knop, Burcak Yesildag, Joachim Størling

**Affiliations:** 1 Translational Type 1 Diabetes Research, Department of Clinical and Translational Research, Steno Diabetes Center Copenhagen, Herlev, Denmark; 2 InSphero AG, Schlieren, Switzerland; 3 Department of Biomedical Sciences, Faculty of Health and Medical Sciences, University of Copenhagen, Copenhagen, Denmark; 4 Novo Nordisk Foundation Center for Basic Metabolic Research, Faculty of Health and Medical Sciences, University of Copenhagen, Copenhagen, Denmark; 5 Center for Clinical Metabolic Research, Gentofte Hospital, University of Copenhagen, Hellerup, Denmark; 6 Department of Clinical Medicine, Faculty of Health and Medical Sciences, University of Copenhagen, Copenhagen, Denmark; 7 Present Address: Novo Nordisk A/S, Søborg, Denmark

**Keywords:** GIP, GLP-1, Glucagon secretion, Human model, Incretins, Liraglutide, Pancreatic alpha cells, Proinflammatory cytokines, Type 1 diabetes

## Abstract

**Aims/hypothesis:**

In type 1 diabetes, the counterregulatory glucagon response to low plasma glucose is impaired. The resulting increased risk of hypoglycaemia necessitates novel strategies to ameliorate alpha cell impairment. Here, we aimed to establish an in vitro type 1 diabetes-like model of alpha cell impairment using standardised reaggregated human islet microtissues (MTs) exposed to proinflammatory cytokines. Additionally, we investigated the therapeutic potential of incretin receptor agonists in improving alpha cell responses to low glucose.

**Methods:**

Human islet MTs were exposed to proinflammatory cytokines (IL-1β, IFN-γ and TNF-α) for 1 day (short-term) and 6 days (long-term). Alpha cell function was assessed by sequential glucose-dependent secretion assays at 2.8 and 16.7 mmol/l glucose, followed by glucagon measurements. Additional evaluations included ATP content, caspase-3/7 activity, chemokine secretion and content of islet transcription factors (aristaless-related homeobox [ARX] and NK6 homeobox 1 [NKX6.1]) and hormones. The effects of incretin receptor agonist treatment (glucose-dependent insulinotropic polypeptide [GIP] analogue [d-Ala^2^]-GIP with or without the glucagon-like peptide 1 [GLP-1] receptor agonist liraglutide) alongside or after cytokine exposure were also investigated, focusing on low-glucose-dependent glucagon secretion.

**Results:**

Short-term cytokine exposure increased glucagon secretion at both 2.8 and 16.7 mmol/l glucose in islet MTs. In contrast, long-term cytokine exposure caused dose-dependent suppression of glucagon secretion at 2.8 mmol/l glucose, resembling a type 1 diabetes phenotype. Long-term cytokine exposure also diminished insulin and somatostatin secretion, reduced ATP content, increased caspase 3/7 activity and decreased islet content of ARX, NKX6.1, glucagon and insulin. Despite cytokine-induced impairment, alpha cells partially retained secretory capacity to l-arginine stimulation. Treatment with incretin receptor agonists during long-term cytokine exposure did not prevent alpha cell impairment. However, acute treatment with [d-Ala^2^]-GIP with or without liraglutide, or with the single-molecule dual agonist tirzepatide, after cytokine exposure partially restored glucagon secretion at low glucose.

**Conclusions/interpretation:**

Long-term cytokine exposure of human islet MTs created a type 1 diabetes-like phenotype with impaired low-glucose-induced glucagon secretion. This cytokine-induced alpha cell impairment was partially restored by [d-Ala^2^]-GIP with or without liraglutide, or by tirzepatide.

**Graphical Abstract:**

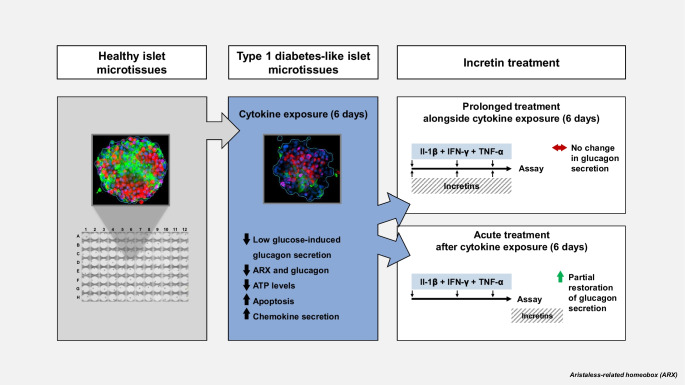

**Supplementary Information:**

The online version of this article (10.1007/s00125-025-06425-3) contains peer-reviewed but unedited supplementary material.


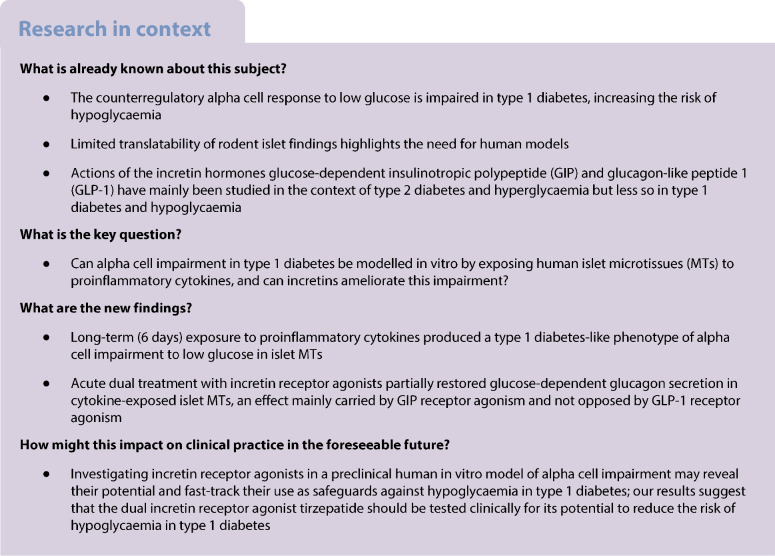


## Introduction

The alpha cells of the pancreatic islets of Langerhans play a pivotal role in glucose homeostasis by secreting counterregulatory glucagon in response to low glucose levels, and this stimulates hepatic glucose output to prevent hypoglycaemia [1]. In individuals with type 1 diabetes, this glucose-dependent counterregulatory alpha cell response is impaired, while their responses to arginine persist, as shown more than 50 years ago by Gerich and coworkers [2]. Consequently, individuals with type 1 diabetes are at an increased risk of hypoglycaemia and, despite significant advances in insulin therapy, hypoglycaemia remains a critical challenge in type 1 diabetes management by limiting proper glycaemic control and by contributing to morbidity and mortality [3]. Despite the crucial role of glucagon in blood glucose regulation, the mechanisms underlying glucose-dependent regulation of glucagon secretion in health and disease remain incompletely understood [4]. Efforts to elucidate alpha cell impairment in the context of type 1 diabetes are hampered by the limited availability of human islets from individuals with type 1 diabetes [5], highlighting a need for establishing models of alpha cell impairment.

Exposure of islets to proinflammatory cytokines is widely used to mimic immune-mediated beta cell destruction [6–8]. However, whether cytokine-exposed islets can also serve as a valid model of alpha cell dysfunction has not been established. Human islets exhibit considerable heterogeneity, adding to the challenge of studying alpha cell responses in vitro [9, 10]. However, recent advances have led to development of reaggregated islet models, known as islet microtissues (MTs). Islet MTs are highly homogenous in size and cell composition thereby strongly limiting within-donor variation [11, 12]. Islet MTs are therefore a valuable tool for investigating islet function in a standardised setting, enhancing the ability to draw conclusions while maintaining the physiological relevance inherent to human donors [11, 12]. This is particularly relevant in the context of preclinical testing of novel therapeutic interventions [13].

Glucagon-like peptide 1 (GLP-1) and glucose-dependent insulinotropic polypeptide (GIP) play essential roles in glucose homeostasis through their glucose-dependent effects on islet insulin and glucagon secretion [14, 15]. GLP-1 receptor agonists are extensively used in the treatment of type 2 diabetes and obesity and are also emerging as a promising therapy to maintain residual beta cell function in newly diagnosed type 1 diabetes [16, 17]. The glucagonotropic effect of GIP at low plasma glucose levels holds promise as a means of reducing the risk of hypoglycaemia in type 1 diabetes [18], though GIP alone may not be sufficiently effective [19]. Single-molecule dual GIP and GLP-1 receptor agonists are currently under extensive development, with tirzepatide already approved to treat type 2 diabetes [15, 20]. Tirzepatide was recently shown to reduce elevated fasting plasma glucagon in type 2 diabetes [21], but tirzepatide’s direct effect on alpha cells particularly in the context of type 1 diabetes remains unclear. Furthermore, species-specific differences in receptor engagement underscore the necessity of using human models to accurately evaluate their therapeutic efficacy and potential in type 1 diabetes [22, 23].

Here, we used human islet MTs exposed to proinflammatory cytokines to create a type 1 diabetes-like model of alpha cell dysfunction. We further evaluated the therapeutic potential of single and dual incretin receptor agonists to evaluate their therapeutic potential for improving alpha cell function.

## Methods

### Human islet MTs

Human islet MTs were acquired from InSphero (Zurich, Switzerland) [11, 12]. Islet MTs from a total of 13 donors (11 male sex, two female sex, mean ± SD age 50±11 years, mean ± SD BMI 29±3.7 kg/m^2^, mean ± SD HbA_1c_ 34±3.7 mmol/mol [5.3±0.3%]) were used. All islet MTs were sourced from non-diabetic, anonymised and cadaveric organ donors. Informed consent was obtained at the isolation site according to local ethical legislation. Donor details are summarised in Table 1. Islet MTs were maintained in 3D InSight Human Islet Maintenance Medium (InSphero) at 37°C in a humidified 5% CO_2_ incubator, with the culture medium renewed every 2–3 days. All experiments and test conditions were performed unmasked to the experiment conductor(s).

**Table 1:** Human islet donor details [55]

Donor	Figures	Unique identifier	Age (years)	Sex	BMI (kg/m^2^)	HbA_1c_ (mmol/mol) (%)	Source of islet MTs	Islet isolation centre	History of diabetes	Summary data included in ESM
1	Fig. 1b and ESM Figs 1a, 2d	AKAR092	55	Male	30.4	38 (5.6)	InSphero	Prodo	No	Yes (ESM Fig. 6a, p, r)
2	Fig. 1c and ESM Figs 1b, 2e	AKBL227	60	Male	32.9	31 (5.0)	InSphero	Prodo	No	Yes (ESM Fig. 6a, p, r)
3	Figs 2b, 3a and ESM Fig. 2a	AJG3470	46	Male	23.1	30 (4.9)	InSphero	Prodo	No	Yes (ESM Fig. 6c, d, q)
4	Figs 2c, d, 3b and ESM Figs 2b, 3a–j	AJHU080	51	Male	31.3	37 (5.5)	InSphero	Prodo	No	Yes (ESM Fig. 6b–d, q)
5	Figs 2e, f, 3c and ESM Fig. 2c	AJIS384	67	Male	34.9	34 (5.3)	InSphero	Prodo	No	Yes (ESM Fig. 6b–d, q)
6	ESM Fig. 2f	AJHR202	54	Male	28.2	39 (5.7)	InSphero	Prodo	No	Yes (ESM Fig. 6r)
7	Fig. 9c, d, i, j and ESM Fig. 2g	AKCE427	48	Male	23.7	37 (5.5)	InSphero	Prodo	No	Yes (ESM Fig. 6l–o, r)
8	Figs 3d, 6, 7b, c, 9b and ESM Fig. 4	AJI4346	37	Male	29.1	36 (5.4)	InSphero	Prodo	No	No
9	Figs 4a–d, 5a–e	AKJG366	58	Female	31.0	28 (4.7)	InSphero	Prodo	No	Yes (ESM Fig. 6e–k)
10	Figs 4e–h, 5f–j	ALG3464	56	Female	25.2	32 (5.1)	InSphero	Prodo	No	Yes (ESM Fig. 6e–k)
11	Fig. 5k–o	AKJ1361	47	Male	28.1	34 (5.3)	InSphero	Prodo	No	Yes (ESM Fig. 6h–k)
12	Figs 7e–h, 8a–j, 9g, h and ESM Fig. 5a–j	AJKO093	32	Male	26.9	30 (4.9)	InSphero	Prodo	No	Yes (ESM Fig. 6n, o)
13	Fig. 9e, f	AKHX448	33	Male	33.1	39 (5.7)	InSphero	Prodo	No	Yes (ESM Fig. 6l, m)

Based on the ‘checklist for reporting human islet preparations used in research’ (see ESM) adapted from [55]

### Cytokine treatment

Islet MTs were cultured for 1 day (short-term) or 6 days (long-term) with or without a mixture of recombinant human IL-1β (R&D Systems, USA or Sigma-Aldrich, USA), recombinant human IFN-γ (PeproTech, USA or R&D Systems) and recombinant human TNF-α (R&D Systems) in a 1:5:5 ratio. A range of cytokine concentrations were tested (ESM Table 1). For the long-term experiments, the culture medium with or without cytokines was renewed every 2–3 days.

### Glucose-dependent secretion assays and hormone quantification

Islet MTs were incubated sequentially in 3D InSight Krebs-Ringer HEPES buffer (KRHB; InSphero) with 0.5% wt/vol. BSA (Sigma-Aldrich) with low glucose (2.8 mmol/l) and high glucose (16.7 mmol/l) (Sigma-Aldrich) for 2 h each. Before initiating a secretion assay, islet MTs were washed twice, equilibrated for 1 h and washed again in 2.8 mmol/l glucose KRHB. Supernatant fractions were collected and immediately frozen until hormone quantification. Glucagon was quantified using the V-PLEX Metabolic Panel 1 Human Kit (with only glucagon capture antibody; Meso Scale Diagnostics, USA) on a MESO Quickplex SQ 120 instrument (Meso Scale Diagnostics) and insulin was quantified using the Stellux Chemi Human Insulin ELISA (Alpco, USA) with a Spark 10M microplate reader (Tecan, Switzerland) according to the manufacturer’s instructions. Somatostatin was measured by RIA as previously described [24] using antibody 1758. Technical replicates were pooled to achieve sufficient sample volume required for accurate measurement of somatostatin.

### Accumulated hormones and chemokines in the culture medium

Culture medium samples collected just prior to glucose-dependent secretion assays were used to determine ‘accumulated’ glucagon and chemokine secretion over 24 h. Briefly, samples were immediately frozen upon collection and later quantified using the V-PLEX Metabolic Panel 1 Human Kit (with only glucagon capture antibody) and V-PLEX Chemokine Panel 1 Human Kit, respectively, on the MESO Quickplex SQ 120 instrument (all Meso Scale Diagnostics). The chemokine panel included chemokine (C-X-C motif) ligand (CXCL) 8 and 10, and C-C motif chemokine ligand (CCL) 2, 3, 4, 11, 13, 17, 22 and 26. Sample values below the limit of detection (LOD) were imputed with the LOD/√2 method [25].

### ATP and apoptosis measurements

In a subset of experiments, MTs were lysed after KRHB sample collection to determine ATP content using the CellTiter-Glo Luminescent Cell Viability Assay (with protease inhibitor cocktail, both from Promega, USA). Luminescence was recorded using the Spark 10M microplate reader (Tecan). Apoptosis was determined as caspase-3/7 activity immediately after the 6 day culture using Caspase-Glo 3/7 Assay (Promega). Luminescence was recorded with the Infinite M200 PRO microplate reader (Tecan).

### Immunofluorescence staining

Islet MTs were immunofluorescently stained for the beta and alpha cell transcription factors NK6 homeobox 1 (NKX6.1) and aristaless-related homeobox (ARX), respectively, and for the three major islet hormones (insulin, glucagon and somatostatin). Briefly, islet MTs were fixed for 15 min using 4% wt/vol. paraformaldehyde (Thermo Fisher Scientific, USA) in PBS (Sigma-Aldrich) and permeabilised with 0.5% vol./vol. Triton X-100 (Sigma-Aldrich) in PBS. Blocking was performed for 1 h using 5% vol./vol. donkey serum (DKS; Jackson ImmunoResearch, UK) for ARX/NKX6.1 staining or 10% vol./vol. FCS (Bio&Sell, Germany) for islet hormone staining. Following blocking, islet MTs were incubated overnight with primary antibodies in antibody incubation buffer (0.2% vol./vol. Triton X-100 in PBS with 5% vol./vol. DKS or 10% vol./vol. FCS), followed by a 4 h secondary antibody incubation in antibody incubation buffer with DAPI nuclear stain. After both antibody incubations, islet MTs were washed with 0.2% vol./vol. Triton X-100 in PBS to eliminate non-specific binding. Antibody and DAPI details are listed in ESM Table 2.

### Confocal imaging and quantitative analysis

Post staining, islet MTs were cleared using ScaleS4 (40% wt/vol. d-(–)sorbitol (PanReac AppliChem, Darmstadt, Germany), 24% wt/vol. urea (Sigma-Aldrich), 8% vol./vol. glycerol (Sigma-Aldrich), 0.2% vol./vol. Triton X-100 and 15% vol./vol. DMSO (PanReac AppliChem) in MilliQ water [26]) and imaged in Akura 384 ImagePro plates (InSphero) using the CQ1 benchtop high content analysis system (Yokogawa, Japan) with a ×40 dry objective (numerical aperture [NA] 0.95). Images were acquired at a z-step size of 3 µm and analysed with the CQ1 analysis software CellPathFinder version 3.07.01.06 (Yokogawa), using a customised pipeline to quantify DAPI-positive, ARX and NKX6.1 mean intensities or to quantify DAPI-positive, glucagon, insulin and somatostatin mean intensities.

### Arginine stimulation and incretin receptor agonists

For the arginine stimulation test, 10 mmol/l l-arginine (Sigma-Aldrich) was added acutely in the glucose-dependent secretion assay. Effects of the incretins [d-Ala^2^]-GIP (a stable GIP analogue) and liraglutide (a long-acting GLP-1 receptor agonist) (both from Tocris Bioscience, UK), alone and in combination, were investigated by adding them alongside the 6 day cytokine exposure, each at a concentration of 1 μmol/l. In a subset of these experiments, incretin treatment was extended to include a 24 h preincubation period and was also added during the glucose-dependent secretion assay. The effects of the [d-Ala^2^]-GIP liraglutide and the dual incretin receptor agonist tirzepatide (Selleck Chemicals, USA) were further investigated by adding them acutely during glucose-dependent secretion assays post 6 day cytokine exposure.

### Statistical analyses

Statistical analyses of non-randomised data were performed using GraphPad Prism v10 (GraphPad, USA). Data are presented as mean ± SD for technical replicates (one donor per graph, Table 1) unless otherwise stated. Outliers were identified and omitted using the robust regression and outlier removal test (ROUT), with the Q-cutoff set to 5%. Comparisons between two sample groups were analysed with unpaired Student’s *t*-tests, whereas comparisons between more than two sample groups were analysed by unpaired one-way or two-way ANOVA with Dunnett’s, Tukey’s or Šídák’s multiple comparison tests, as indicated in the figure legends. Statistical significance was defined as a *p* value <0.05.

## Results

### Short-term cytokine exposure increases glucagon secretion

**Fig. 1:**
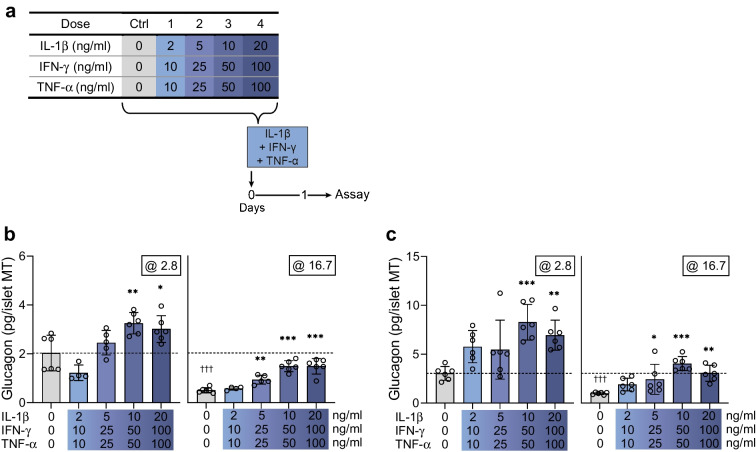
Short-term cytokine exposure increases glucagon secretion. (**a**) Experimental schematic of the short-term (1 day) setup. (**b**, **c**) Glucagon secretion at 2.8 and 16.7 mmol/l glucose in control islet MTs (grey bars) and with increasing load of short-term cytokine exposure (blue bars) in donor 1 (**b**) and donor 2 (**c**). The dashed line denotes the baseline physiological response to low glucose of untreated control islet MTs. Data are presented as mean ± SD of a single donor in six technical replicates. *p<0.05, **p<0.01 and ***p<0.001 vs untreated control, by one-way ANOVA with Dunnett’s multiple comparisons test; ^†††^*p*<0.001, for the two untreated controls at 2.8 vs 16.7 mmol/l glucose, by Student’s *t* test

We subjected human islet MTs from two donors to short-term (1 day) cytokine (IL-1β + IFN-γ + TNF-α) exposure, followed by sequential glucose-dependent secretion assays (Fig. 1a). Cytokine exposure caused a dose-dependent increase in glucagon secretion compared with untreated control MTs at both low (2.8 mmol/l) and high (16.7 mmol/l) glucose (Fig. 1b, c). Insulin secretion was mainly affected by cytokines at low glucose (ESM Fig. 1). These results show that short-term cytokine exposure leads to aberrant glucagon and insulin secretion but not in a pattern resembling that found in type 1 diabetes.

### Long-term cytokine exposure decreases glucagon secretion

**Fig. 2:**
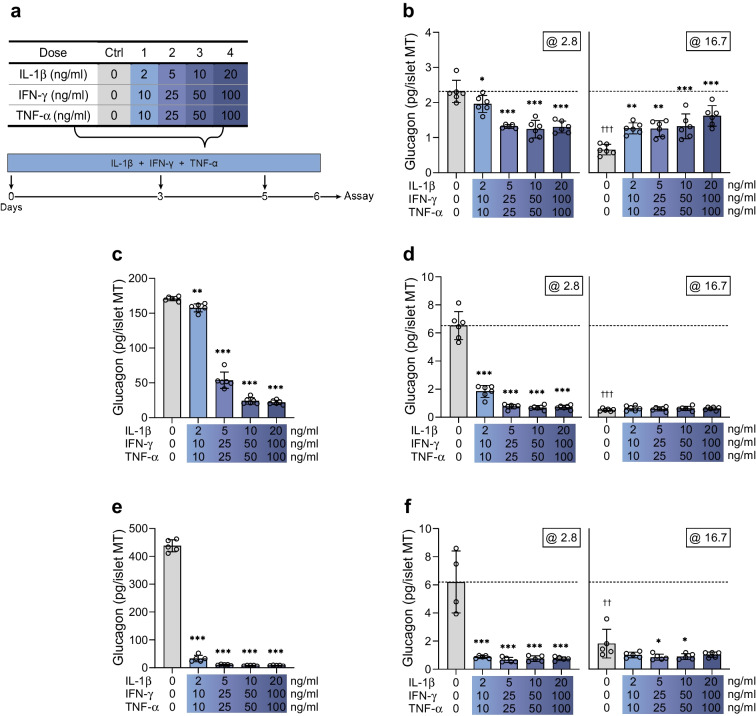
Long-term cytokine exposure impairs glucose-dependent glucagon secretion. (**a**) Experimental schematic of the long-term (6 day) setup. (**b**, **d**, **f**) Glucagon secretion at 2.8 and 16.7 mmol/l glucose in control islet MTs (grey bars) and with increasing load of long-term cytokine exposure (blue bars) in donor 3 (**b**), donor 4 (**d**) and donor 5 (**f**). The dashed line denotes the baseline physiological response to low glucose of untreated control islet MTs. (**c**, **e**) Accumulated glucagon secretion over the last 24 h of long-term cytokine exposure for donor 4 (**c**) and donor 5 (**e**). Data are presented as mean ± SD of a single donor in six technical replicates (five technical replicates for donor 5). *p<0.05, **p<0.01 and ***p<0.001 vs untreated control, by one-way ANOVA with Dunnett’s multiple comparisons test; ^††^*p*<0.01 and ^†††^*p*<0.001, for the two untreated controls at 2.8 vs 16.7 mmol/l glucose, by Student’s *t* test

We next extended cytokine exposure to 6 days (long-term) before assessing the secretory function (Fig. 2a). We found that long-term cytokine exposure diminished glucagon secretion in response to low glucose for all tested cytokine doses in islet MTs from three donors (Fig. 2b, d, f). Accumulated glucagon secretion during the last 24 h of cytokine exposure was also decreased (Fig. 2c, e). Baseline glucagon secretion and cytokine sensitivity were highly donor dependent. The cytokine-induced impairment of low-glucose-induced glucagon secretion was in the range 15.5–43.0% (Fig. 2b), 71.9–89.2% (Fig. 2d) and 86.1–88.0% (Fig. 2f) for donor 3, donor 4 and donor 5, respectively. In islet MTs from one donor, the 6 day cytokine exposure exacerbated glucagon secretion at high glucose (92.0–146.4%, Fig. 2b), whereas it was unaffected or reduced in the two other donors (Fig. 2d, f). The detrimental effects of long-term cytokine exposure on high-glucose-induced insulin secretion were more consistent among the three donors, with reductions in the range 28.8–89.3%, 20–63.7% and 24.4–92.1%, respectively (ESM Fig. 2a–c). Somatostatin secretion was generally decreased at both low and high glucose (ESM Fig. 2d–g). These data demonstrate that long-term cytokine exposure leads to impaired alpha cell function.

### Long-term cytokine exposure increases cell death and chemokine secretion, and reduces cell identity markers

**Fig. 3:**
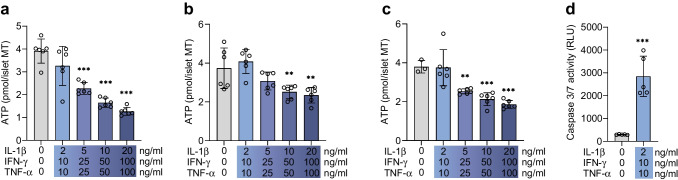
Long-term cytokine exposure induces cell death. (**a**–**c**) ATP content in control (grey bars) and with increasing load of long-term cytokine exposure (blue bars) in donor 3 (**a**), donor 4 (**b**) and donor 5 (**c**). Data are presented as mean ± SD of a single donor in six technical replicates. **p<0.01 and ***p<0.001 vs untreated control, by one-way ANOVA with Dunnett’s multiple comparisons test. (**d**) Caspase 3/7 activity after long-term cytokine exposure (dose 1, donor 8). Data are presented as mean ± SD of a single donor in five technical replicates. ***p<0.001, for untreated control vs cytokine exposure, by Student’s *t* test. RLU, relative light units

**Fig. 4:**
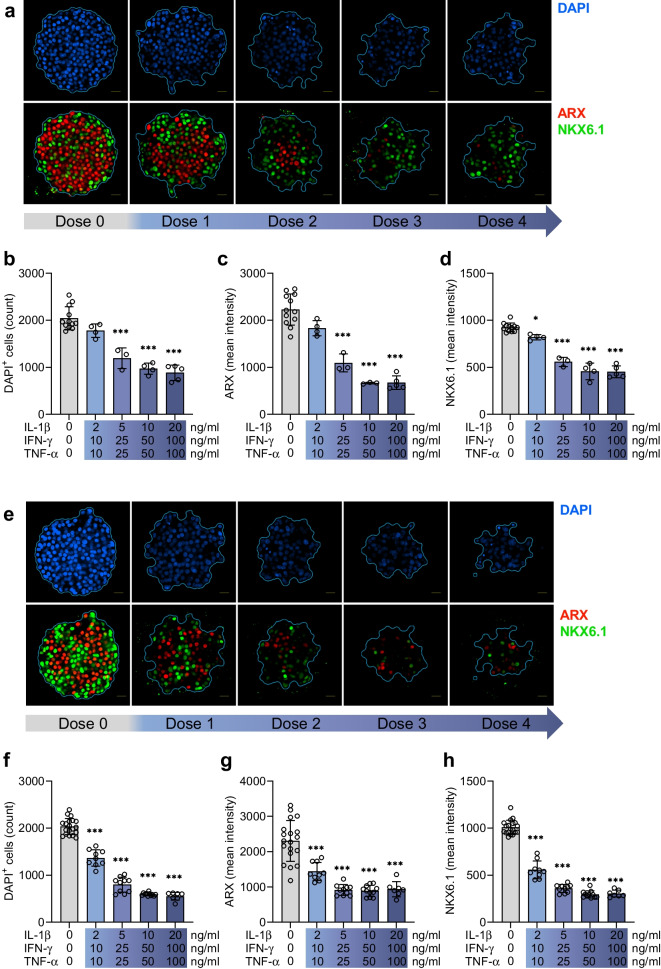
Long-term cytokine exposure reduces transcription factor ARX and NKX6.1 expression. (**a**–**d**) Representative images for transcription factor staining in islet MTs from donor 9 with increasing load of long-term cytokine exposure (**a**) and associated quantification of DAPI-positive cell count (**b**), mean ARX intensity (**c**) and mean NKX6.1 intensity (**d**) expression. (**e**–**h**) Representative images for transcription factor staining in islet MTs from donor 10 (**e**) and associated quantification of DAPI-positive cell count (**f**), mean ARX intensity (**g**) and mean NKX6.1 intensity (**h**). Scale bar, 20 μm. Data are presented as mean ± SD of a single donor in 3–11 technical replicates (12–19 for control). *p<0.05 and ***p<0.001 vs untreated control, using one-way ANOVA with Dunnett’s multiple comparisons test

**Fig. 5:**
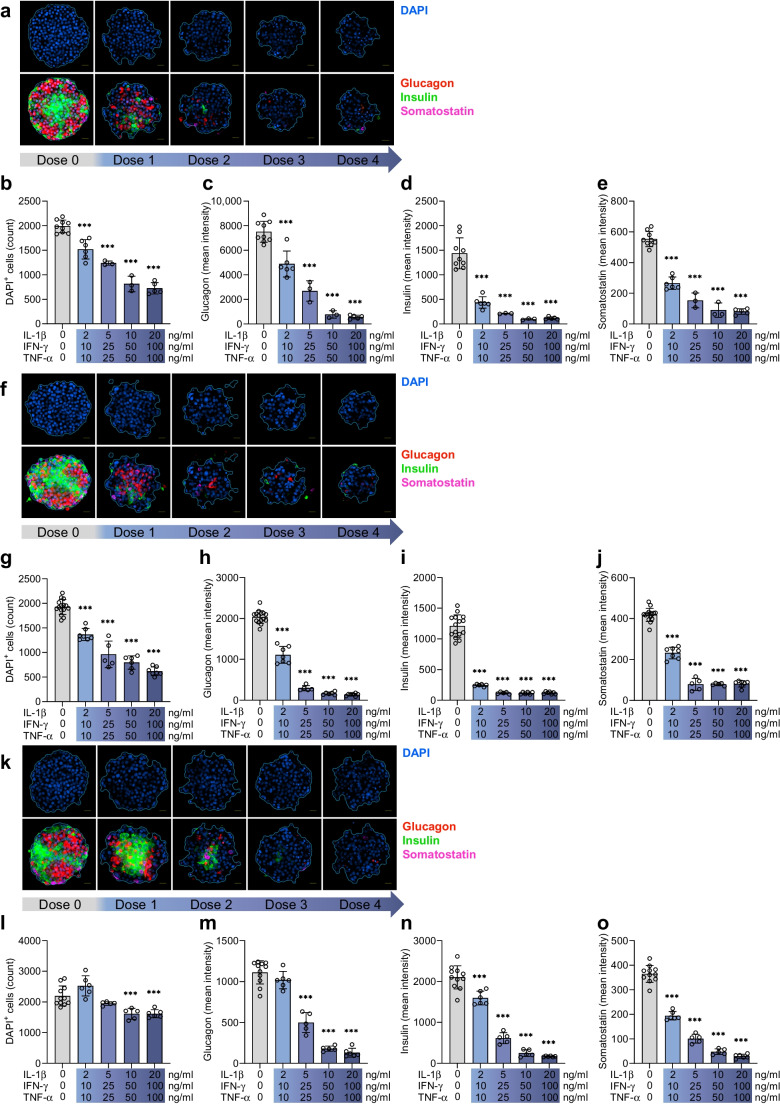
Long-term cytokine exposure reduces islet hormone expression. (**a**–**e**) Representative images for islet hormone staining in islet MTs from donor 9 with increasing load of long-term cytokine exposure (**a**) and associated quantifications of DAPI-positive cell count (**b**), mean glucagon intensity (**c**), mean insulin intensity (**d**) and mean somatostatin intensity (**e**). (**f**–**j**) Representative images for islet hormone staining in islet MTs from donor 10 (**f**) and associated quantifications of DAPI-positive cell count (**g**), mean glucagon intensity (**h**), mean insulin intensity (**i**) and mean somatostatin intensity (**j**). (**k–o**) Representative images for islet hormone staining in islet MTs from donor 11 (**k**) and associated quantifications of DAPI-positive cell count (**l**), mean glucagon intensity (**m**), mean insulin intensity (**n**), and mean somatostatin intensity (**o**). Scale bar, 20 μm. Data are presented as mean ± SD of a single donor in 3–7 technical replicates (9–15 for control). ***p<0.001 vs untreated control, by one-way ANOVA with Dunnett’s multiple comparisons test

To further assess the functional impact of long-term cytokine treatment on islet MTs, we assessed ATP content, caspase 3/7-activity, DAPI nuclei staining, cell identity markers and secretion of chemokines. In islet MTs from three donors, ATP content dropped dose-dependently in response to cytokines (Fig. 3a–c). Apoptosis was increased, reflected by increased caspase 3/7 activity following cytokine exposure of islet MTs from one donor (Fig. 3d). In line with this, cytokine-treated islet MTs from three donors showed dose-dependent reductions in the DAPI-positive cell count (Figs 4a, b, e, f, 5a, b, f, g, k, l). Immunofluorescence staining of the alpha cell marker ARX and beta cell marker NKX6.1 in two donors showed dose-dependent reductions in response to cytokines (Fig. 4a, c–e, g, h). Accordingly, glucagon, insulin, and somatostatin were dose-dependently reduced in response to cytokines in three donors (Fig. 5a, c–e, f, h–j, k, m–o). Cytokine exposure also increased the secretion of several chemokines (ESM Fig. 3).

### Alpha cells partially retain secretory capacity after long-term cytokine exposure

**Fig. 6:**
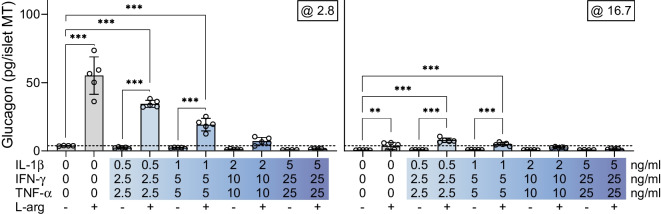
Alpha cells partially retain secretory capacity in response to l-arginine after long-term cytokine exposure. Glucagon secretion at 2.8 and 16.7 mmol/l glucose in control (grey bars) and with increasing load of long-term cytokine exposure (blue bars) in islet MTs from donor 8 with and without 10 mmol/l l-arginine. The dashed line denotes the baseline physiological response to low glucose of untreated control islet MTs. Data are presented as mean ± SD of five technical replicates. **p<0.01 and ***p<0.001, for l-arginine stimulated MTs vs the respective unstimulated control and for l-arginine stimulated MTs vs the untreated control/baseline response, by two-way ANOVA with Šídák’s multiple comparisons test, L-arg, l-arginine

We next investigated whether the alpha cells retain capacity to secrete glucagon in response to l-arginine following long-term cytokine exposure, as seen in type 1 diabetes [2]. As the highest doses of cytokines used so far exerted very harsh effects on the islet MTs, we substituted these with lower doses to optimise the experimental window (ESM Fig. 4, ESM Table 1). In untreated control islet MTs, l-arginine induced a 1355% increase in glucagon secretion at low glucose (Fig. 6). In MTs exposed to the two lowest cytokine doses, l-arginine significantly increased glucagon to an extent surpassing that of low glucose alone in control MTs (dotted line, Fig. 6). l-arginine produced only modest effects at high glucose. These results demonstrate that alpha cells partially retain the ability to secrete glucagon in response to l-arginine after long-term cytokine exposure.

### Treatment with GIP and/or GLP-1 receptor agonists alongside cytokines does not prevent cytokine-induced detrimental effects

**Fig. 7:**
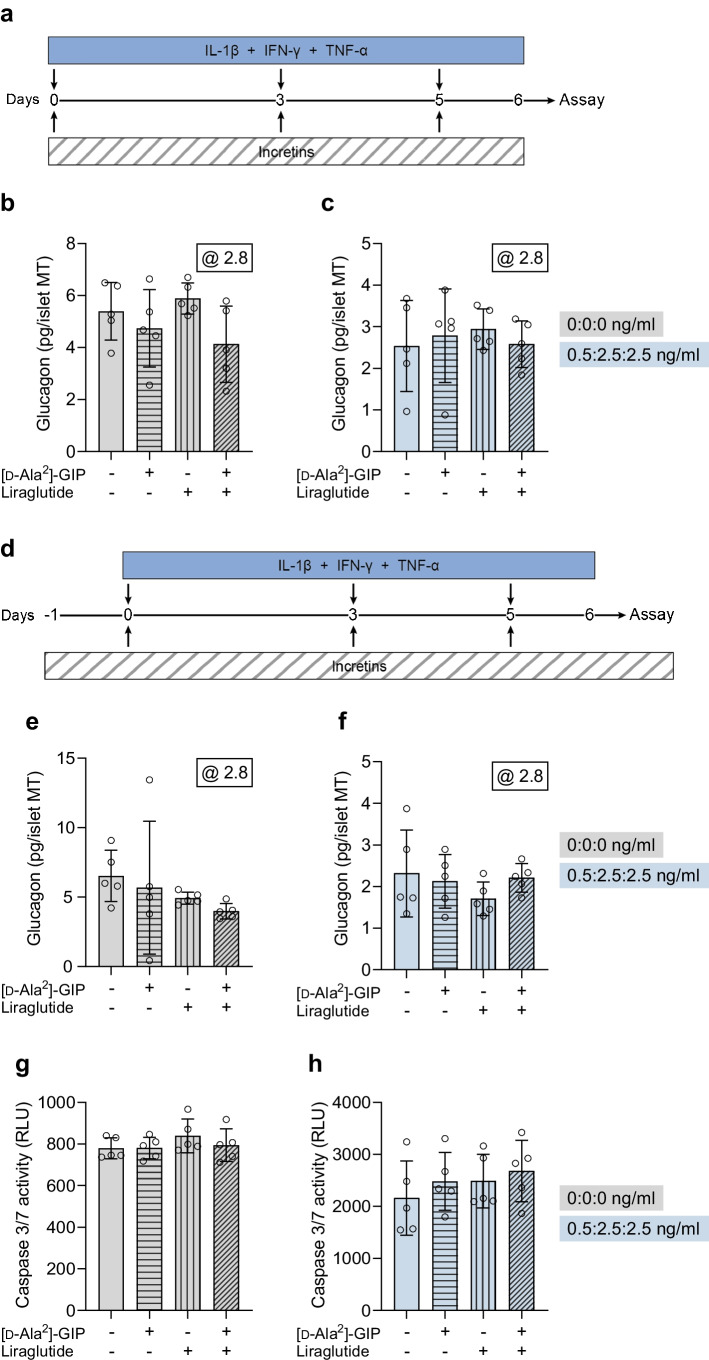
Treatment with [d-Ala^2^]-GIP with or without liraglutide alongside cytokine exposure does not prevent alpha cell impairment or cell death. (**a**) Experimental schematic for donor 8. (**b**, **c**) Glucagon secretion in islet MTs from donor 8 at 2.8 mmol/l glucose in control MTs (**b**), and long-term cytokine-exposed MTs (at dose ¼: 0.5, 2.5 and 2.5 ng/ml for IL-1β, IFN-γ and TNF-α, respectively) (**c**), without or with 1 µmol/l [d-Ala^2^]-GIP, liraglutide, or both. (**d**) Experimental schematic for donor 12 denoting addition of 24 h pre-treatment with [d-Ala^2^]-GIP and/or liraglutide before cytokine exposure and the addition during glucose-dependent hormone secretion assay. (**e**, **f**) Glucagon secretion in islet MTs from donor 12 at 2.8 mmol/l glucose in control MTs (no cytokines) (**e**), and long-term cytokine-exposed MTs (at dose ¼: 0.5, 2.5 and 2.5 ng/ml for IL-1β, IFN-γ and TNF-α, respectively) (**f**), without or with 1 µmol/l [d-Ala^2^]-GIP, liraglutide, or both. (**g**, **h**) Caspase 3/7 activity in islet MTs from donor 12 in control islet MTs (no cytokines) (**g**), and after long-term cytokine exposure (dose ¼: 0.5, 2.5 and 2.5 ng/ml for IL-1β, IFN-γ and TNF-α, respectively) (**h**) without or with 1 µmol/l [d-Ala^2^]-GIP, liraglutide, or both. Data are presented as mean ± SD of a single donor in five technical replicates. Results are not significant using one-way ANOVA with Tukey’s multiple comparisons test. RLU, relative light units

Incretin receptor agonists exert anti-inflammatory and cytoprotective effects on human islets [27, 28], and liraglutide improves beta cell function of islet MTs under cytokine conditions [7]. We therefore investigated whether treatment with GIP and/or GLP-1 receptor agonists during the long-term low-dose cytokine exposure would prevent alpha cell impairment (Fig. 7a). Long-term treatment with the stable GIP analogue [d-Ala^2^]-GIP, the long-acting GLP-1 receptor agonist liraglutide, or a combination of the two, alongside cytokines had no effects on glucagon secretion at low glucose (Fig. 7b, c). The inclusion of a 24 h pre-treatment step with [d-Ala^2^]-GIP with or without liraglutide before cytokine exposure and inclusion of these during the glucose-dependent secretion assay (Fig. 7d) still failed to alter glucagon secretion (Fig. 7e, f).

**Fig. 8:**
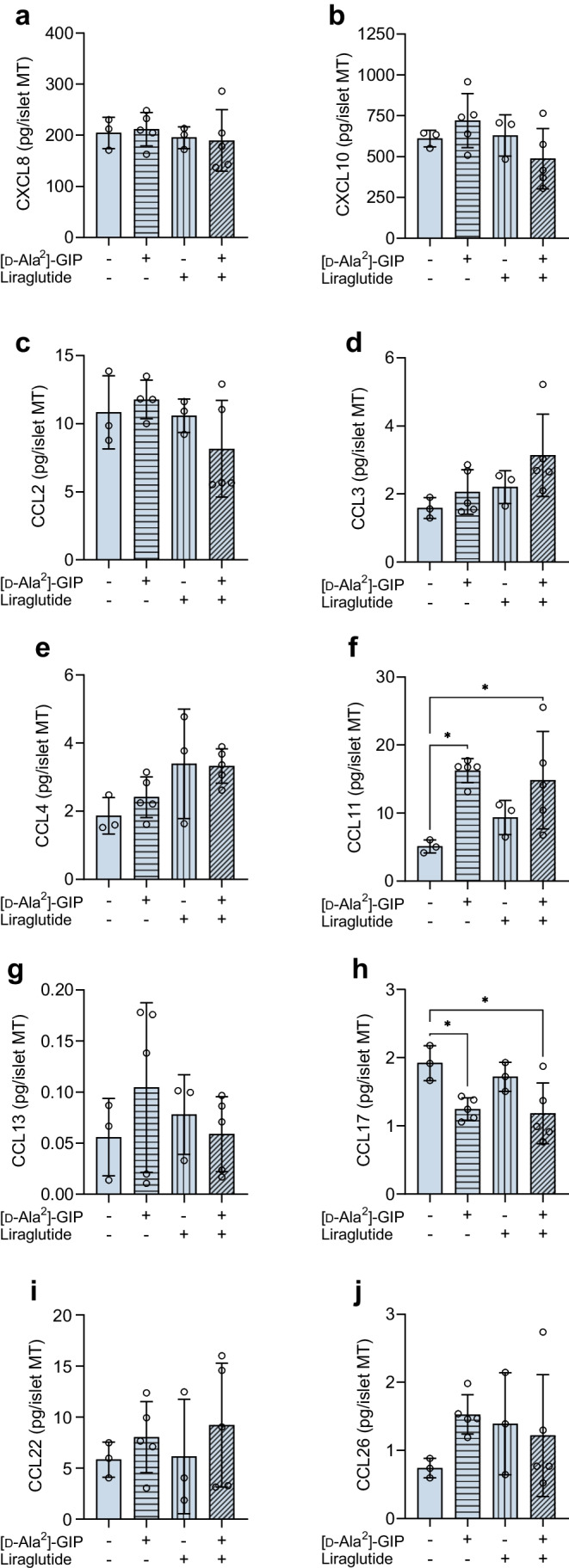
Treatment with [d-Ala^2^]-GIP with or without liraglutide alters cytokine-induced secretion of CCL11 and CCL17. Accumulated CXCL8 (**a**), CXCL10 (**b**), CCL2 (**c**), CCL3 (**d**), CCL4 (**e**), CCL11 (**f**), CCL13 (**g**), CCL17 (**h**), CCL22 (**i**) and CCL26 secretion (**j**) in islet MTs from donor 12 during the last 24 h of the long-term cytokine exposure (dose ¼) without or with 1 µmol/l [d-Ala^2^]-GIP, liraglutide, or both. Data are presented as mean ± SD in 5 technical replicates (only three technical replicates were measured for untreated control and liraglutide bars due to pipetting error). *p<0.05, for all pairwise comparisons between treatment groups, by one-way ANOVA with Tukey’s multiple comparisons test

Consistent with these findings, long-term treatment with incretin receptor agonists alongside cytokine exposure failed to prevent cytokine-induced caspase 3/7 activity (Fig. 7g, h). Analysis of chemokine secretion during the last 24 h of the long-term exposure showed that while the secretion of chemokines was induced by cytokine exposure (compared with baseline levels; ESM Fig. 5), only two cytokine-induced chemokines were modulated by [d-Ala^2^]-GIP with/without liraglutide treatment (Fig. 8). Specifically, [d-Ala^2^]-GIP with and without liraglutide increased CCL11 secretion (Fig. 8f*)* while decreasing CCL17 secretion (Fig. 8h). The obtained data indicate that at the tested doses, treatment with incretin receptor agonists alongside cytokine exposure fails to prevent the detrimental effects of cytokines on glucagon secretion and cell death, but modestly alter the secretion of chemokines.

### Acute treatment with GIP and/or GLP-1 receptor agonists after cytokine exposure partially restores glucose-dependent glucagon secretion

**Fig. 9:**
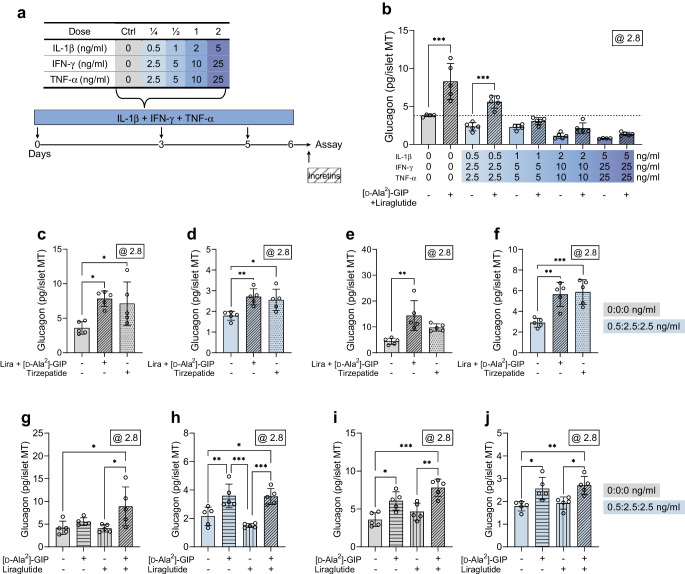
Acute treatment with incretins following cytokine exposure boosts glucagon secretion of the type 1 diabetes phenotype. (**a**) Experimental schematic denoting acute incretin treatment during the hormone secretion assay. (**b**) Glucagon secretion in islet MTs from donor 8 at 2.8 mmol/l glucose in control islet MTs or in the islet MTs with increasing load of long-term cytokine exposure, with/without acute treatment with [d-Ala^2^]-GIP+liraglutide (1 µmol/l each) during the hormone secretion assay. (**c**, **d**) Glucagon secretion in islet MTs from donor 7 at 2.8 mmol/l glucose in control (**c**) and cytokine-exposed islet MTs (dose ¼: ¼: 0.5, 2.5 and 2.5 ng/ml for IL-1β, IFN-γ and TNF-α, respectively) (**d**) without or with acute [d-Ala^2^]-GIP+liraglutide (1 µmol/l each) or 1 µmol/l tirzepatide treatment. (**e**, **f**) As for (**c**, **d**) but in islet MTs from donor 13. (**g**, **h**) Glucagon secretion in islet MTs from donor 12 at 2.8 mmol/l glucose in control (**g**) and cytokine-exposed islet MTs (dose ¼: ¼: 0.5, 2.5 and 2.5 ng/ml for IL-1β, IFN-γ and TNF-α, respectively) (**h**) with/without acute treatment with 1 µmol/l [d-Ala^2^]-GIP, liraglutide, or both. (**i**, **j**) As for (**g**, **h**) but in islet MTs from donor 7. Data are presented as mean ± SD of a single donor in five technical replicates. *p<0.05, **p<0.01 and ***p<0.001, for [d-Ala^2^]-GIP+liraglutide treatment vs the respective untreated control, by two-way ANOVA with Šídák’s multiple comparisons test (**b**); *p<0.05, **p<0.01 and ***p<0.001, for [d-Ala^2^]-GIP+liraglutide or tirzepatide treatment vs the untreated control, by one-way ANOVA with Dunnett’s multiple comparisons test (**c**–**f**); *p<0.05, **p<0.01 and ***p<0.001, for all pairwise comparisons between treatment groups, by one-way ANOVA with Tukey’s multiple comparisons test (**g**–**j**). Ctrl, control

Next, we tested the effects on glucagon secretion of [d-Ala^2^]-GIP with and without liraglutide treatment in islet MTs after long-term cytokine exposure (Fig. 9a). Acute treatment with [d-Ala^2^]-GIP plus liraglutide following cytokine exposure yielded a 119% increase in baseline low-glucose-induced glucagon secretion (Fig. 9b). A comparable increase was observed for islet MTs exposed to the lowest cytokine dose (140%), with the alleviation surpassing the baseline glucagon secretion response to low glucose (dashed line, Fig. 9b). At higher cytokine doses, [d-Ala^2^]-GIP plus liraglutide no longer boosted glucagon secretion. In a subset of experiments, we included the single-molecule dual GIP receptor and GLP-1 receptor agonist tirzepatide (Fig. 9c–f). Similar to the effect observed for [d-Ala^2^]-GIP plus liraglutide, tirzepatide treatment augmented glucagon secretion in both untreated control (Fig. 9c, e) and cytokine-exposed (Fig. 9d, f) islet MTs from two donors.

Finally, we examined the individual contribution made by [d-Ala^2^]-GIP and liraglutide to the observed partial restoration of low-dose cytokine-induced alpha cell impairment (Fig. 9g–j). [d-Ala^2^]-GIP alone or in combination with liraglutide yielded similar increases in glucagon secretion in islet MTs from two donors (Fig. 9h, j). Liraglutide alone did not significantly affect glucagon secretion.

## Discussion

In this study, we aimed to establish an in vitro type 1 diabetes-like model of human alpha cell impairment using islet MTs exposed to cytokines. Our data demonstrate that 6 days of cytokine exposure leads to alpha cell impairment, which can be partly restored by acute treatment with [d-Ala^2^]-GIP with or without liraglutide, or by tirzepatide.

Short-term (1 day) cytokine exposure increased glucagon secretion dose-dependently at both low and high glucose. Although postprandial (i.e. high glucose) glucagon hypersecretion has been described early in type 1 diabetes disease progression [29], short-term cytokine exposure did not appear to reflect a type 1 diabetes phenotype with regards to the hypoglycaemic state (low glucose), characterised by reduced glucagon secretion [2, 30, 31]. This increase in glucagon secretion may reflect an initial response to cytokines, as increased glucagon has been shown to correlate with inflammation [32]. Alternatively, the increased glucagon secretion may reflect alpha cell dysfunction preceding beta cell loss, as described for autoantibody-positive donors [33]. This is further supported by glucose-stimulated insulin secretion being only modestly altered at high glucose in these experiments. Indeed, while reduced glucose-stimulated insulin secretion has been observed in rodent models in the time range of 24–48 h, human islets appear to require longer exposure times [34]. This is supported by recent studies using islet MTs, showing that prolonged cytokine challenge reduced glucose-stimulated insulin secretion [6, 7].

Our data show that prolonged (6 days) cytokine exposure suppresses the function of alpha, beta and delta cells. We observed donor-specific variations in the sensitivity of individual cell types to the detrimental effects of cytokines. This is not surprising considering the normal heterogeneity of native human islets from which the MTs were generated [9, 10]. Despite donor-to-donor variability, the overall effects observed were consistent across multiple experiments (see ESM Fig. 6 for summary data for experiments performed on two or more donors). Immunofluorescence staining supported the notion that cytokines ‘hit’ the alpha cells, as evidenced by reduced glucagon staining intensity at the lowest cytokine dose used in two out of three donors tested. However, insulin (and somatostatin) staining intensity was decreased in all three donors. Some cells with intact nuclei but that were hormone-negative remained after cytokine exposure. This suggests that prolonged cytokine exposure may trigger islet cell dedifferentiation to escape cell death. In line with this, cytokines suppress the expression of islet identity genes in human islets [35], and an increase in hormone-negative endocrine cells is seen in islets from donors with type 1 diabetes [36]. Our staining experiments also indicate donor-dependent variation in MT alpha cell and beta cell populations, with alpha cells occasionally being as abundant as beta cells. Similarly, immunostaining of human islets, as opposed to murine islets, showed considerable endocrine cell population variability, with alpha cell proportions averaging 34% but ranging from 10% to 65% [9].

Several putative mechanisms behind the impaired counterregulatory glucagon secretion in type 1 diabetes have been proposed. Most likely, impairment cannot be ascribed to a single responsible factor or mechanism but is the consequence of an interplay between several factors related to paracrine signalling (e.g. loss of intra-islet insulin or increased somatostatin secretion) [37–39]. Despite the strongly impaired low-glucose-induced glucagon secretion, the ability to respond to l-arginine was partially maintained, especially in islet MTs exposed to the lower range of cytokine doses, suggesting the presence of alpha cells with secretory capacity exceeding basal low-glucose secretion and an assay window for translational exploration of augmentation of glucagon responses. Following the initial experiments establishing the effects of cytokines on MTs (Figs 1, 2, 3, 4, and 5), we introduced lower cytokine doses in the experiments with l-arginine and when testing the incretin receptor agonists in order to obtain MTs more mildly affected by cytokines and thereby create a better window of opportunity.

The pathophysiology of type 1 diabetes is characterised by islet inflammation, and both beta cells [40] and alpha cells [41] play an active role during type 1 diabetes progression (e.g. via secretion of chemokines with immune-attractant effects). Consistent with this, we found that the secretion of nine chemokines was increased upon long-term cytokine exposure. Hence, islet MTs, similar to native human islets and beta cell models, secrete chemokines in response to cytokines. Also consistent with type 1 diabetes pathophysiology, our model demonstrated dose-dependent reductions in ATP content and number of DAPI-positive cells, and increased caspase 3/7 activity. We cannot, however, associate these effects to specific islet cell types. Based on the reduction in cell identity markers and hormones, it is plausible that the ATP content was decreased, and death increased in alpha, beta and delta cells in this model. Although this differs from type 1 diabetes in situ, where the beta cells are exclusively destroyed, we believe that the cytokine–MT model has value as a human ex vivo system to study dysfunctional alpha cells and act as a testing platform for new alpha cell-boosting treatments.

We and others have described GIP as a dual-acting hormone with both insulinotropic and glucagonotropic effects in isolated human islets [42, 43], in healthy individuals [14] and in individuals with type 1 diabetes [18]. GLP-1 receptor agonists have been described in pre-translational beta cell models as anti-apoptotic and are reported to prevent loss of glucose-stimulated insulin secretion [7, 28]. We hypothesised that dual incretin receptor agonism would be superior to GIP receptor agonism alone in preventing the cytokine-induced type 1 diabetes alpha cell phenotype indirectly via preservation of beta cells and/or beta cell function. Contrary to this hypothesis, treatment with [d-Ala^2^]-GIP plus liraglutide alongside cytokine exposure did not prevent alpha cell impairment, and liraglutide alone did not reduce apoptosis in the tested conditions. No protective effects of liraglutide in terms of reduced secretion of chemokines were observed, as only [d-Ala^2^]-GIP alone significantly changed the secretion of two chemokines (CCL11 and CCL17), though in the opposite directions. The lack of effect of liraglutide could, at least in part, be explained by liraglutide’s palmitic acid chain that binds to albumin for stability, which necessitates high concentrations for in vitro experiments to have an adequate available pool of liraglutide for receptor binding [44]. Exceeding the 1 μmol/l concentration used here may enhance liraglutide’s functional impact. In the experiments with treatment alongside cytokine exposure, [d-Ala^2^]-GIP alone did not prevent the development of the type 1 diabetes phenotype of alpha cells either. However, GIP receptor desensitisation due to chronic GIP presence has been reported in mouse adipocytes [45], and human GIP receptors appear to be more prone to desensitisation through β-arrestin recruitment [46]. Hence, the lack of an alpha cell-preservative effect in our study could be due to the chronic nature of [d-Ala^2^]-GIP treatment, causing desensitisation of the GIP receptor.

Notably, we found that acute [d-Ala^2^]-GIP treatment augmented glucagon secretion in untreated control islet MTs and partially restored glucagon secretion after long-term cytokine exposure. Interestingly, despite reports of glucagonostatic effects of GLP-1 on glucagon secretion at low glucose [47, 48], [d-Ala^2^]-GIP plus liraglutide was comparable with [d-Ala^2^]-GIP alone in augmenting glucagon secretion. Furthermore, liraglutide alone did not inhibit glucagon secretion. GLP-1’s effects on alpha cells are complex and probably mainly indirect, as GLP-1 receptor expression is thought to be limited or absent in human alpha cells, although uncertainty exists [47, 49]. However, there is some evidence that GLP-1 may act directly on alpha cells, as glucagonostatic effects are independent of insulin and somatostatin, as evidenced by insulin receptor and somatostatin receptor 2 blockage [47]. In any case, the glucagonostatic effect at low glucose remains controversial, as the glucagonostatic effects of GLP-1 receptor agonists have mainly been described during fasting hyperglycaemia or postprandial hyperglycaemia as in type 2 diabetes [50, 51]. In addition, several studies have found that GLP-1 receptor agonists did not compromise the hypoglycaemic glucagon response in healthy individuals and individuals with type 1 diabetes [52, 53]. The ambiguous findings in the literature underline the complexity of incretin–islet biology, demanding further studies, especially in human islet model systems.

Importantly, we found comparable effects of [d-Ala^2^]-GIP plus liraglutide and tirzepatide regarding low-glucose-induced glucagon secretion. This observation is supported by the apparent main contribution of [d-Ala^2^]-GIP in driving the glucagonotropic effect when in combination with liraglutide, and the fact that tirzepatide has been reported to be an imbalanced GIP/GLP-1 receptor agonist (i.e. higher receptor engagement at the GIP receptor than at the GLP-1 receptor) and biased towards cAMP generation at the GLP-1 receptor [22, 54]. Additionally, while the glucagonotropic effect of tirzepatide during low glucose has been established [22], we now extend this to a model of type 1 diabetes alpha cell impairment.

In summary, this study presents an in vitro model of a type 1 diabetes-like alpha cell phenotype obtained by exposure to proinflammatory cytokines. The model enables functional studies and screening of compounds to resurrect glucose-dependent glucagon secretion. Our study also shows for the first time that [d-Ala^2^]-GIP can ameliorate alpha cell impairment to low glucose after cytokine exposure, which was not opposed by liraglutide. Additionally, tirzepatide, recognised as an imbalanced incretin co-agonist, can ameliorate alpha cell impairment to low glucose following cytokine exposure. Our findings should be considered in the light of limitations inherent in human donor studies, specifically stemming from the restricted pool of donors.

## Supplementary Information

Below is the link to the electronic supplementary material.

ESM (PDF 1364 KB)

## Data Availability

The datasets generated and analysed during the study are available from the corresponding author upon reasonable request.

## Abbreviations

ARX: Aristaless-related homeobox
CCL: C-C Motif chemokine ligand
CXCL: Chemokine (C-X-C motif) ligand
GIP: Glucose-dependent insulinotropic polypeptide
GLP-1: Glucagon-like peptide 1
KRHB: Krebs-Ringer HEPES buffer
LOD: Limit of detection
MT: Microtissue
NKX6.1: NK6 homeobox 1

## Authors’ relationships and activities

CR, ACT, SJ and BY are employees of InSphero. BH is a co-founder of Bainan Biotech. JJH has served on scientific advisory panels for and/or has received speaker honoraria from Novo Nordisk and MSD/Merck, is co-founder and board member of Antag Therapeutics, and is co-founder of Bainan Biotech. FKK has served on scientific advisory panels and/or been part of speaker’s bureaus for, served as a consultant to, and/or received research support from Amgen, AstraZeneca, Bayer, Boehringer Ingelheim, Carmot Therapeutics, Eli Lilly, Gubra, MedImmune, MSD/Merck, Mundipharma, Norgine, Novo Nordisk, Sanofi, ShouTi, Zealand Pharma and Zucara. FKK is a co-founder of and minority shareholder in Antag Therapeutics, owns stocks in Eli Lilly, Novo Nordisk and Zealand Pharma, and has been employed by Novo Nordisk since 1 December 2023. JS owns stocks in Novo Nordisk A/S. The authors declare that there are no other relationships or activities that might bias, or be perceived to bias, their work.
