# Supplementary material for: Proinflammatory cytokine-induced alpha cell impairment in human islet microtissues is partially restored by dual incretin receptor agonism
Source: Diabetologia. 2025 May 15;68(7):1492–508. doi: 10.1007/s00125-025-06425-3 (PMC12176928; doi:10.1007/s00125-025-06425-3)
Supplement: Supplementary file 1 — ESM (PDF 1364 KB) [file 125_2025_6425_MOESM1_ESM.pdf]

Electronic Supplementary Material (ESM)

Overview

ESM Tables **p. 2**

ESM Table 1 Related to Methods. Cytokines and doses.

ESM Table 2 Related to Methods. Antibodies and dilutions used for immunofluorescent staining and confocal imaging.

ESM Figures **p. 4**

ESM Fig. 1 Related to Fig. 1

ESM Fig. 2 Related to Fig. 2

ESM Fig. 3 Related to Fig. 3

ESM Fig. 4 Related to Fig. 6

ESM Fig. 5 Related to Fig. 8

ESM Fig. 6 Summary data of experiments with data from ≥2 donors.

ESM Checklists **p. 13**

Checklist for reporting human islet preparations used in research

ESM Tables

ESM Table 1 Related to Methods. Cytokines and doses.

	Cytokine	Company Cat. No.	Dose 0 (ng/ml)	Dose ¼ (ng/ml)	Dose ½ (ng/ml)	Dose 1 (ng/ml)	Dose 2 (ng/ml)	Dose 3 (ng/ml)	Dose 4 (ng/ml)
Set 1	IL-1β	Sigma-Aldrich, USA #H6291-10UG	0			2	5	10	20
	IFN-γ	R&D systems, USA #10067-IF-100	0			10	25	50	100
	TNF-α	R&D systems, USA #10291-TA-050	0			10	25	50	100
Set 2	IL-1β	R&D systems, USA #201-LB-005	0	0.5	1	2	5		
	IFN-γ	Peprotech, USA #300-02	0	2.5	5	10	25		
	TNF-α	R&D systems, USA #210-TA-005	0	2.5	5	10	25		

ESM Table 2 Related to Methods. Antibodies and dilutions used for immunofluorescent staining and confocal imaging.

Antibody	Supplier, #Cat. number	Dilution
Primary antibodies		
Sheep anti-ARX	R&D Systems, USA (#AF7068-SP)	1:400
Rabbit anti-NKX6.1	Abcam, UK (#AB221549)	1:200
Mouse anti-Glucagon	Sigma-Aldrich, USA (#G2654)	1:300
Mouse anti-Insulin (AF488)	Invitrogen, USA (#53-9769-82)	1:100
Rat anti-Somatostatin	Invitrogen, USA (#MA5-16987)	1:200
Secondary antibodies		
Donkey anti-sheep (AF647)	Jackson ImmunoResearch, UK (#713-605-147)	1:200
Donkey anti-rabbit (AF568)	Invitrogen, USA (#A10042)	1:200
Alpaca anti-mouse (AF568)	ChromoTek, GER (#sms1AF568-1-100)	1.200
Goat anti-rat (AF647)	Jackson Immunoresearch, UK (#112-605-167)	1:100
Fluorescent stains		
DAPI	Sigma-Aldrich, USA (#D9542)	1:500

ESM Figures

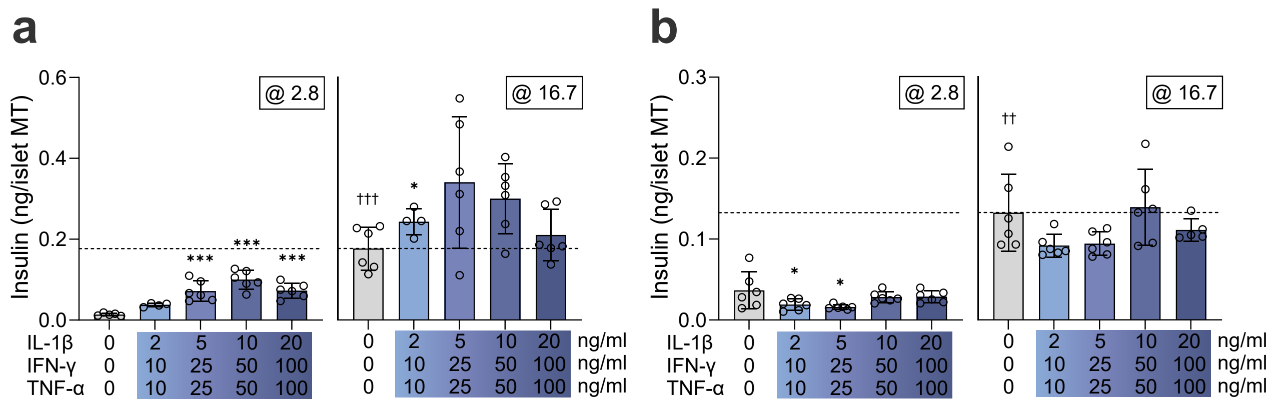

ESM Fig. 1 Related to Fig. 1. (**a, b**) Insulin secretion at 2.8 and 16.7 mmol/l glucose in control islet MTs (grey bars) and with increasing load of short-term cytokine exposure (blue bars) in donor 1 (**a**) and donor 2 (**b**). The dashed line denotes the baseline physiological response to high glucose of untreated control islet MTs. Data are presented as mean ± SD of a single donor in six technical replicates. **p*<0.05, ****p*<0.001 vs untreated control, by one-way ANOVA with Dunnett's multiple comparisons test; ††*p*<0.01, †††*p*<0.001 for the two untreated controls at 2.8 vs 16.7 mmol/l glucose, by Student's *t* test.

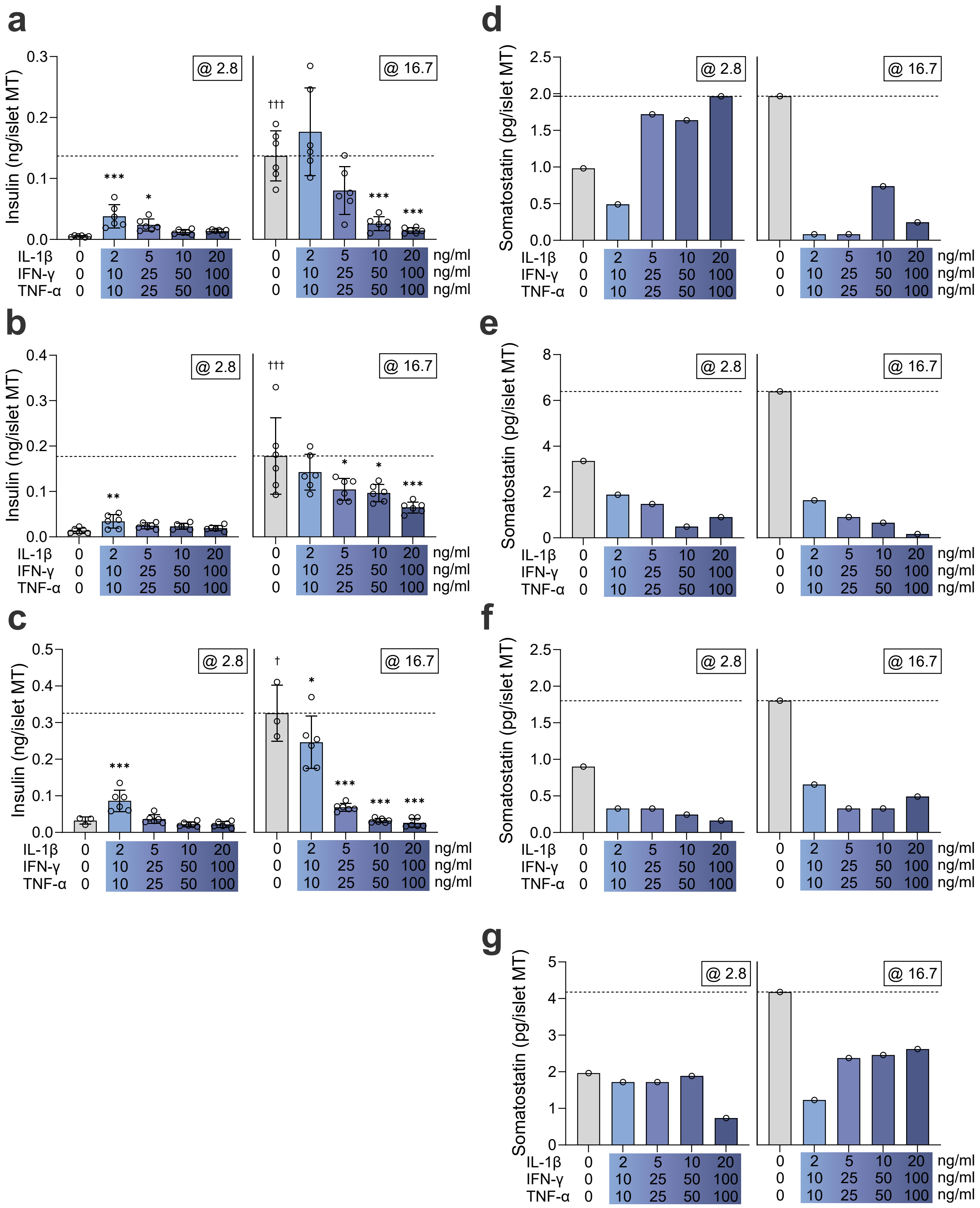

ESM Fig. 2 Related to Fig. 2. (**a-c**) Insulin secretion at 2.8 and 16.7 mmol/l glucose in control islet MTs (grey bars) and with increasing load of long-term cytokine exposure (blue bars) in donor 3 (**a**), donor 4 (**b**), and

donor 5 (**c**). The dashed line denotes the baseline physiological response to high glucose of untreated control. (**d-g**) Somatostatin secretion at 2.8 and 16.7 mmol/l glucose in control islet MTs (grey bars) and with increasing load of long-term cytokine exposure (blue bars) in donor 1 (**d**), donor 2 (**e**), donor 6 (**f**), and donor 7 (**g**). Data in (**a-c**) are presented as mean ± SD of a single donor in six technical replicates. *p<0.05, **p<0.01, ***p<0.001 vs untreated control, by one-way ANOVA with Dunnett's multiple comparisons test; †p<0.05, †††p<0.001 for the two untreated controls at 2.8 vs 16.7 mmol/l glucose, by Student's t test. Data in (**d–g**) represent values measured from six pooled technical replicates from a single donor. No statistical analysis was applied due to the sample size limitation.

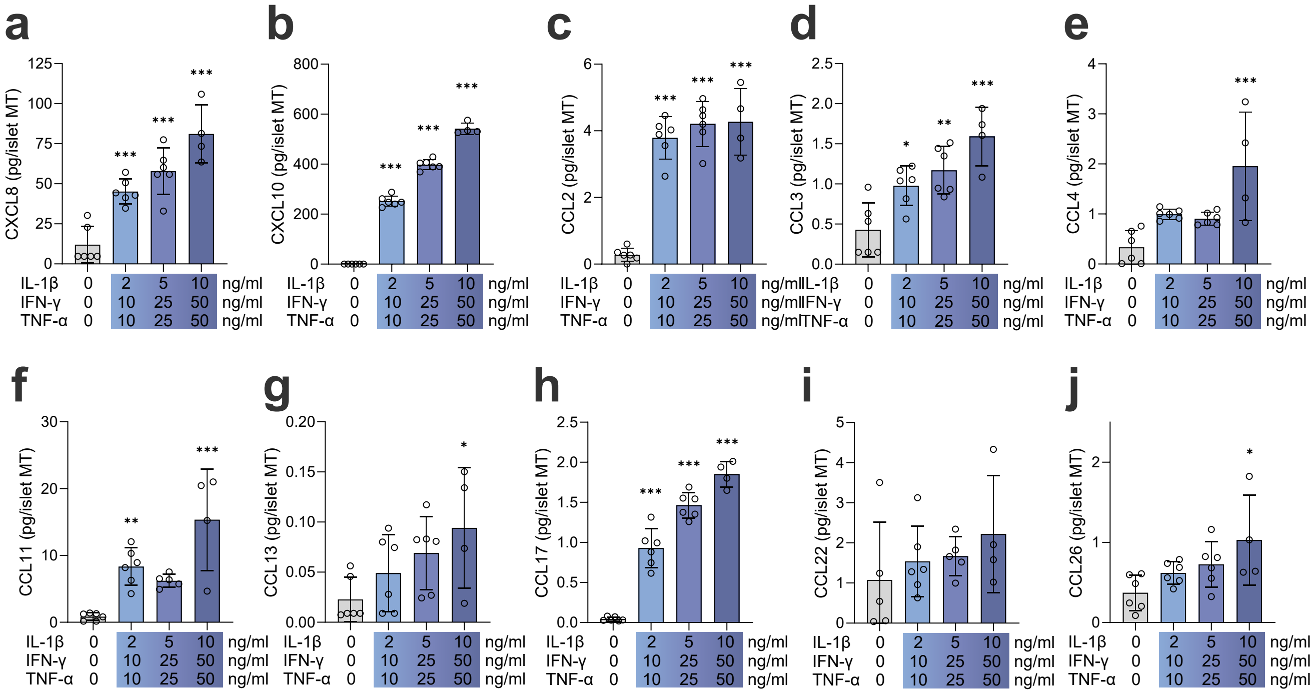

ESM Fig. 3 Related to Fig. 3. Accumulated CXCL8 (**a**), CXCL10 (**b**), CCL2 (**c**), CCL3 (**d**), CCL4 (**e**), CCL11 (**f**), CCL13 (**g**), CCL17 (**h**), CCL22 (**i**), and CCL26 (**j**) secretion during the last 24 hours of increasing long-term cytokine exposure (blue bars) or untreated control (grey bar) in islet MTs from donor 4. Data are presented as mean ± SD of a single donor in six technical replicates (only four technical replicates for dose 3 due to technical error). **p*<0.05, ***p*<0.01, ***p*<0.001 vs untreated control, by one-way ANOVA with Dunnett's multiple comparisons test.

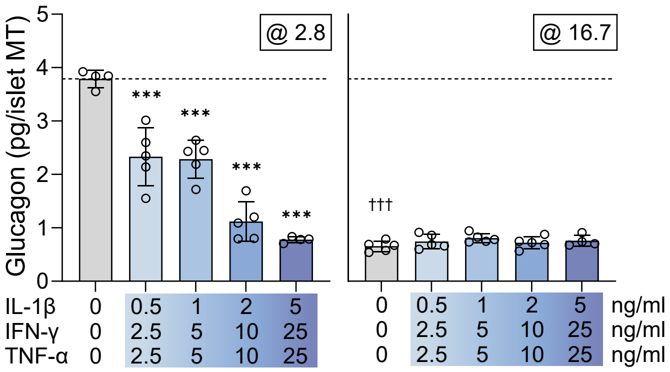

ESM Fig. 4 Related to Fig. 6. Glucagon secretion at 2.8 and 16.7 mmol/l glucose in control islet MTs (grey bars) and following long-term cytokine exposure with optimised dose titration (dose ¼, ½, 1 and 2). The dashed line denotes the untreated control baseline/physiological response to low glucose. Data presented as mean ± SD of donor 8 in five technical replicates. ***p<0.001 vs untreated control, by one-way ANOVA with Dunnett's multiple comparisons test; †††p<0.001 for the two untreated controls at 2.8 vs. 16.7 mmol/l glucose, by Student's t test.

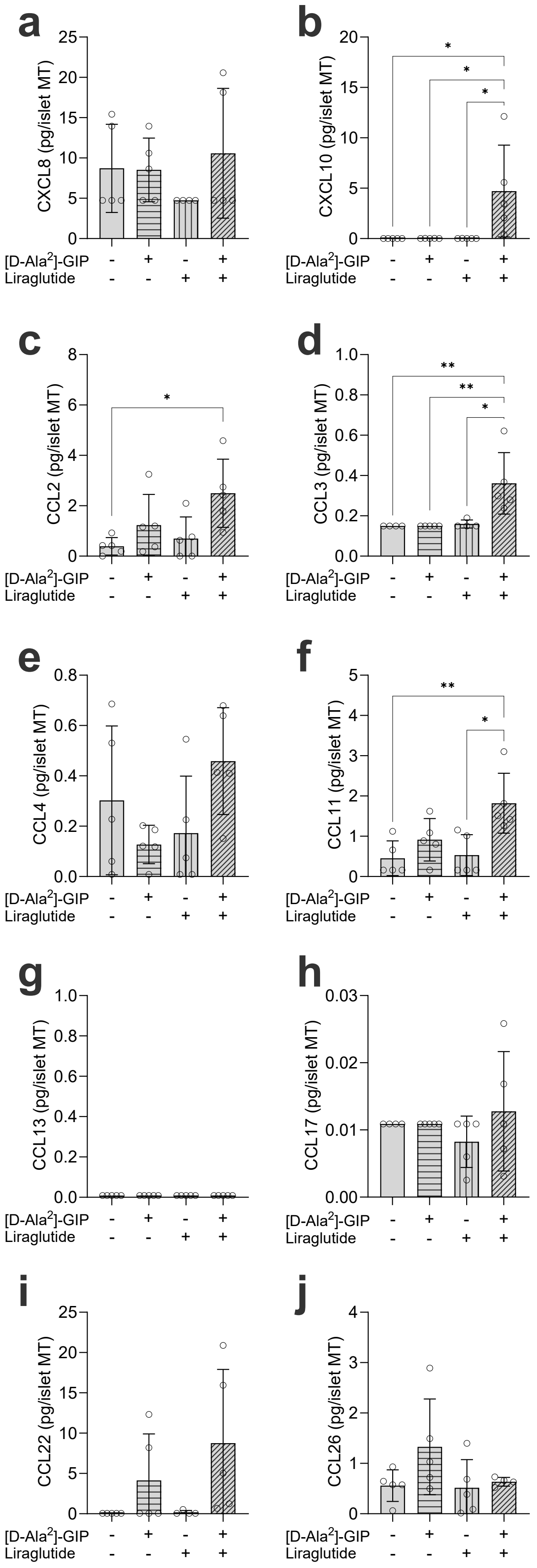

ESM Fig. 5 Related to Fig. 8. Accumulated CXCL8 (**a**), CXCL10 (**b**), CCL2 (**c**), CCL3 (**d**), CCL4 (**e**), CCL11 (**f**), CCL13 (**g**), CCL17 (**h**), CCL22 (**i**), and CCL26 (**j**) secretion during the last 24 h of no cytokine

exposure with or without 1 μmol/l [D-Ala^2]-GIP, liraglutide, or [D-Ala^2]-GIP+liraglutide. Data are presented as mean ± SD of donor 12 in five technical replicates. *$p<0.05$, **$p<0.01$, for all pairwise comparisons between treatment groups, by one-way ANOVA with Tukey's multiple comparisons test.

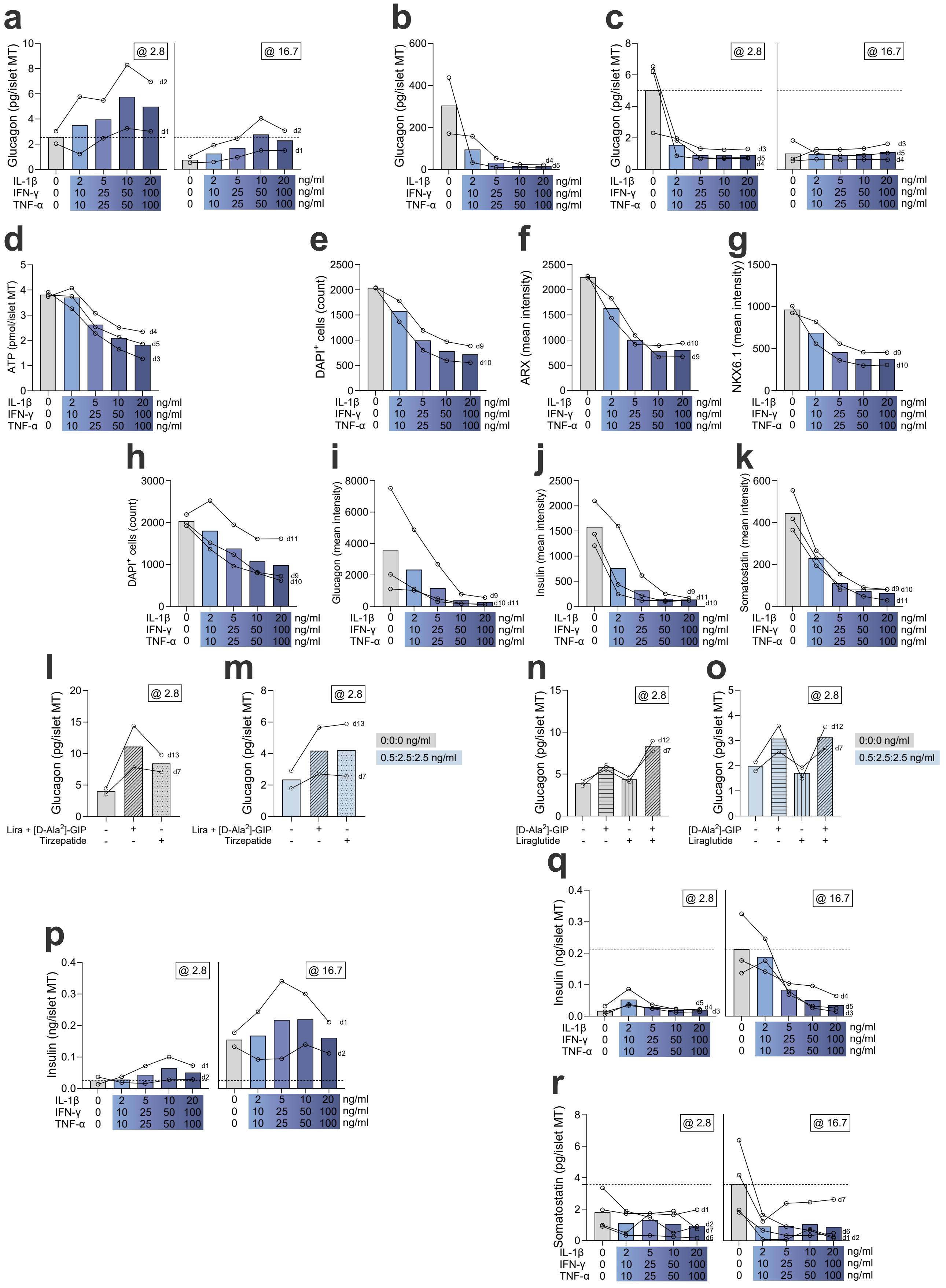

(PREVIOUS PAGE) ESM Fig. 6 Summary data of experiments with data from ≥2 donors. (**a**) Summary data for Fig. 1 Short-term cytokine exposure increases glucagon secretion. (**b, c**) Summary data for Fig. 2 Long-term cytokine exposure impairs glucose-dependent glucagon secretion. (**d**) Summary data for Fig. 3a-c Long-term cytokine exposure induces cell death. (**e-g**) Summary data for Fig. 4 Long-term cytokine exposure reduces transcription factor ARX and NKX6.1 expression (**h-k**) Summary data for Fig. 5 Long-term cytokine exposure reduces islet hormone expression. (**l, m**) Summary data for Fig. 9c-f Acute treatment with incretins following cytokine exposure boosts glucagon secretion of the type 1 diabetes phenotype. (**n, o**) Summary data for Fig. 9g-j Acute treatment with incretins following cytokine exposure boosts glucagon secretion of the type 1 diabetes phenotype. (**p**) Summary data for ESM Fig. 1 Related to Fig. 1. (**q, r**) Summary data for ESM Fig. 2 Related to Fig. 2.

Checklist for reporting human islet preparations used in research

Adapted from Hart NJ, Powers AC (2018) Progress, challenges, and suggestions for using human islets to understand islet biology and human diabetes. Diabetologia https://doi.org/10.1007/s00125-018-4772-2

**Islet preparation**	**1**	**2**	**3**	**4**	**5**	**6**	**7**	**8[a]**
**MANDATORY INFORMATION**								
Unique identifier	AKAR092	AKBL227	AJG3470	AJHU080	AJIS384	AJHR202	AKCE427	AJI4346
Donor age (years)	55	60	46	51	67	54	48	37
Donor sex (M/F)	M	M	M	M	M	M	M	M
Donor BMI (kg/m^2)	30.4	32.9	23.1	31.3	34.9	28.2	23.7	29.1
Donor HbA_{1c} or other measure of blood glucose control	38 mmol/mol (5.6 %)	31 mmol/mol (5.0 %)	30 mmol/mol (4.9 %)	37 mmol/mol (5.5 %)	34 mmol/mol (5.3 %)	39 mmol/mol (5.7 %)	37 mmol/mol (5.5 %)	36 mmol/mol (5.4 %)
Origin/source of islets[b]	InSphero AG, Switzerland	InSphero AG, Switzerland	InSphero AG, Switzerland	InSphero AG, Switzerland	InSphero AG, Switzerland	InSphero AG, Switzerland	InSphero AG, Switzerland	InSphero AG, Switzerland
Islet isolation centre	Prodo Laboratories Inc, USA	Prodo Laboratories Inc, USA	Prodo Laboratories Inc, USA	Prodo Laboratories Inc, USA	Prodo Laboratories Inc, USA	Prodo Laboratories Inc, USA	Prodo Laboratories Inc, USA	Prodo Laboratories Inc, USA
Donor history of diabetes? Please select yes/no from drop down list	No	No	No	No	No	No	No	No
**If Yes, complete the next two lines if this information is available**								
Diabetes duration (years)	N/A	N/A	N/A	N/A	N/A	N/A	N/A	N/A
Glucose-lowering therapy at time of death[c]	N/A	N/A	N/A	N/A	N/A	N/A	N/A	N/A
**RECOMMENDED INFORMATION**								
Donor cause of death								
Warm ischaemia time (h)								
Cold ischaemia time (h)								

Estimated purity (%)								
Estimated viability (%)								
Total culture time (h)[d]								
Glucose-stimulated insulin secretion or other functional measurement[e]								
Handpicked to purity? Please select yes/no from drop down list								
Additional notes								

**Islet preparation**	**9**	**10**	**11**	**12**	**13**	**14**	**15**	**16**
**MANDATORY INFORMATION**								
Unique identifier	AKJG366	ALG3464	AKJ1361	AJKO093	AKHX448			
Donor age (years)	58	56	47	32	33			
Donor sex (M/F)	F	F	M	M	M			
Donor BMI (kg/m^2)	31.0	25.2	28.1	26.9	33.1			
Donor HbA_{1c} or other measure of blood glucose control	28 mmol/mol (4.7 %)	32 mmol/mol (5.1 %)	34 mmol/mol (5.3 %)	30 mmol/mol (4.9 %)	39 mmol/mol (5.7 %)			
Origin/source of islets[b]	InSphero AG, Switzerland	InSphero AG, Switzerland	InSphero AG, Switzerland	InSphero AG, Switzerland	InSphero AG, Switzerland			
Islet isolation centre	Prodo Laboratories Inc, USA	Prodo Laboratories Inc, USA	Prodo Laboratories Inc, USA	Prodo Laboratories Inc, USA	Prodo Laboratories Inc, USA			
Donor history of diabetes? Please select yes/no from drop down list	No	No	No	No	No			

**If Yes, complete the next two lines if this information is available**								
Diabetes duration (years)	N/A	N/A	N/A	N/A	N/A			
Glucose-lowering therapy at time of death[c]	N/A	N/A	N/A	N/A	N/A			
**RECOMMENDED INFORMATION**								
Donor cause of death								
Warm ischaemia time (h)								
Cold ischaemia time (h)								
Estimated purity (%)								
Estimated viability (%)								
Total culture time (h)[d]								
Glucose-stimulated insulin secretion or other functional measurement[e]								
Handpicked to purity? Please select yes/no from drop down list								
Additional notes								

[a]If you have used more than eight islet preparations, please complete additional forms as necessary
[b]For example, IIDP, ECIT, Alberta IsletCore
[c]Please specify the therapy/therapies
[d]Time of islet culture at the isolation centre, during shipment and at the receiving laboratory
[e]Please specify the test and the results
